# Using structural equation modeling in the understanding of functional disability in older adults*

**DOI:** 10.1590/1518-8345.4555.3451

**Published:** 2021-06-28

**Authors:** Darlene Mara dos Santos Tavares, Nayara Gomes Nunes Oliveira, Flavia Aparecida Dias Marmo, Joilson Meneguci

**Affiliations:** 1 Universidade Federal do Triângulo Mineiro, Departamento de Enfermagem em Educação e Saúde Comunitária, Uberaba, MG, Brazil.; 2 Universidade Federal do Triângulo Mineiro, Uberaba, MG, Brazil.

**Keywords:** Aged, Health of the Elderly, Activities of Daily Living, Geriatrics, Statistical Models, Geriatric Nursing, Idoso, Saúde do Idoso, Atividades Cotidianas, Geriatria, Modelos Estatísticos, Enfermagem Geriátrica, Anciano, Salud del Anciano, Actividades Cotidianas, Geriatría, Modelos Estadísticos, Enfermería Geriátrica

## Abstract

**Objective:**

to analyze functional disability and its associated factors among community-dwelling older adults.

**Method:**

a cross-sectional study, conducted with 1,635 older adults distributed in the following age groups: 60 to 69, 70 to 79, and 80 years old or more, living in a health macro-region of the state of Minas Gerais. Descriptive and trajectory analysis was carried out (p<0.05). The parameters were estimated by the Maximum Likelihood method.

**Results:**

the highest percentage was female, with a monthly income of 1 minimum wage and living with a companion. In the age groups from 60 to 69 and from 70 to 79 years old, older adults with a partner predominated; and, among those aged 80 years old or more, widowed individuals prevailed. In the three groups, functional disability occurred hierarchically. Lower schooling, frailty and depressive symptomatology were factors directly associated with functional disability in the advanced activities; frailty and sedentary behavior were directly associated with functional disability in the instrumental activities. In the older adults aged between 60 and 69 years old and from 70 to 79 years old, sedentary behavior was associated with greater dependence on the basic activities.

**Conclusion:**

the expanded understanding of the factors in the functional disability of the older adults, according to age group, helps the health professional in the development of preventive measures for this disease.

## Abstract (pt)

**Objetivo:**

analisar a incapacidade funcional e seus fatores associados entre idosos na comunidade.

**Método:**

estudo transversal, com 1.635 idosos distribuídos nas faixas etárias: 60 a 69, 70 a 79 e 80 ou mais, residentes em uma macrorregião de saúde do Estado de Minas Gerais. Procedeu-se à análise descritiva e de trajetórias (*p*<0,05). Os parâmetros foram estimados pelo método da Máxima Verossimilhança.

**Resultados:**

o maior percentual era do sexo feminino, com renda mensal de 1 salário mínimo e morava acompanhado. Nas faixas etárias de 60 a 69 e 70 a 79 anos predominaram os idosos com companheiro, e entre aqueles com 80 anos ou mais os viúvos. Nos três grupos, a incapacidade funcional se deu de forma hierárquica. A menor escolaridade, a fragilidade e a sintomatologia depressiva foram fatores associados diretamente à incapacidade funcional nas atividades avançadas; a fragilidade e o comportamento sedentário associaram-se diretamente à incapacidade funcional nas atividades instrumentais. Nos idosos com idade entre 60 e 69 anos e 70 a 79 anos, o comportamento sedentário associou-se à maior dependência nas atividades básicas.

**Conclusão:**

a compreensão ampliada dos fatores atuantes na incapacidade funcional dos idosos, segundo faixa etária, auxilia o profissional de saúde no desenvolvimento de medidas preventivas desse agravo.

## Abstract (es)

**Objetivo:**

analizar la incapacidad funcional y sus factores asociados entre adultos mayores de la comunidad.

**Método:**

estudio transversal, con 1.635 adultos mayores distribuidos en rangos etarios: 60 a 69, 70 a 79 y 80 o más, residentes en una macrorregión de salud del Estado de Minas Gerais. Se realizó análisis descriptivo y de trayectoria (p<0,05). Los parámetros se estimaron mediante el método de máxima verosimilitud.

**Resultados:**

el mayor porcentaje fue de sexo femenino, con un ingreso mensual de 1 salario mínimo y vivía acompañado. En los rangos etarios de 60 a 69 y de 70 a 79 años predominaron los adultos mayores con pareja, y entre los de 80 años o más, los viudos. En los tres grupos, la incapacidad funcional se presentó de forma jerárquica. Menor educación, fragilidad y síntomas de depresión fueron factores directamente asociados con la incapacidad funcional en actividades avanzadas; la fragilidad y el sedentarismo se asociaron directamente con la incapacidad funcional en las actividades instrumentales. En los adultos mayores de 60 y 69 años y de 70 a 79 años, el sedentarismo se asoció con una mayor dependencia de las actividades básicas.

**Conclusión:**

una mayor comprensión de los factores que influyen en la incapacidad funcional del adulto mayor, según el rango etario, ayuda a los profesionales de la salud en el desarrollo de medidas preventivas de esta enfermedad.

## Introduction

Among the older adults, functional capacity can be considered both determinant and resulting from their health condition^(^ 1 ^)^, being measured by the activities of daily living (ADLs). These are stratified according to the difficulty, complexity and vulnerability to cognitive alterations, in a triple hierarchy, namely: Basic (BADLs), Instrumental (IADLs) and Advanced (AADLs) activities^(^ 2 ^)^.

The functional capacity of the older adults can be influenced by several factors such as demographic, socioeconomic and behavioral characteristics, health condition, use of health services^(^ 3 ^-^ 4 ^)^; widowhood, advanced age, sedentarism, cognitive decline, history of stroke, and hospitalization in the last 12 months^(^ 1 ^)^.

In studies developed with older Turkish^(^ 5 ^)^ and Norwegian^(^ 6 ^)^ older adults, frailty, excessive time in a seated position, short and/or prolonged sleep, and physical inactivity were associated with functional disability. Among Japanese women, decreased walking speed, comorbidities and pain reports were factors associated with this condition^(^ 7 ^)^. In a meta-analysis, it was observed that low physical performance and reduction of strength and muscle mass were associated with functional dependence^(^ 8 ^)^. It is noteworthy that, in a cohort in Norway, older adults with complex comorbidities have higher risks of dependence in the ADLs and of mortality^(^ 9 ^)^.

Although the literature on the theme is vast, finding studies that described the explanatory factors for the relationship between sociodemographic, clinical and behavioral variables with functional capacity, through models previously tested in mediation analyzes, that is, indirect relationships, was a challenge, what justifies this study. In this perspective, for a better understanding of the event, analyses are necessary that consider the direct and mediation effects, such as structural equation models.

The modeling of structural equations allows the simultaneous analysis of the dependence relationship and interrelation of multiple variables. In addition, it estimates direct effects mediated by other factors that integrate the causal network of outcomes of interest^(^ 10 ^-^ 11 ^)^.

Thus, given the results of the studies with this theme^(^ 1 ^,^ 3 ^-^ 9), it is assumed that functional disability results from sociodemographic, economic, clinical and behavioral characteristics of the older adult. However, there are doubts about which of these factors act directly or through mediation. Considering that age is related to functional decline and that this phenomenon can manifest differently among younger and older aged individuals, it is questioned which of these variables is related to functional disability in the age groups from 60 to 69, 70 to 79, and 80 years old or more.

The objective was to analyze functional disability and its associated factors among community-dwelling older adults.

## Method

A cross-sectional and analytical study, guided by the Strengthening the Reporting of Observational Studies in Epidemiology (STROBE) tool. It was developed in the urban area of a health macro-region of the state of Minas Gerais, composed of three health micro-regions, comprising 27 municipalities. Data collection was carried out from May 2017 to June 2018, through direct interview and physical performance tests.

When calculating the sample size, a prevalence of functional disability in the IADLs of 34.2% was considered^(^ 3 ^)^ with 1.5% precision and a 95% confidence interval, for a finite population of 75,726 older adults, reaching a sample of 1,659 aged individuals.

For selecting the older adults, multiple stage cluster sampling was used. In the first stage, the arbitrary draw of 50% of the census sectors of each municipality of the health macro-region was considered by systematic sampling. For each municipality, the number of households to be selected was calculated, proportionally to the total number of older adults living in the 27 cities of the health macro-region. Subsequently, the number of households was divided by the number of census sectors, obtaining a similar number of older adults to be interviewed within each census sector. Finally, in each census sector, the first household was randomly selected and the others, in a standardized sense, until saturating the sector sample.

The criteria for inclusion were the following: being 60 years of age or older and living in the urban area of the health macro-region. Older adults with cognitive decline were excluded; as well as those with severe stroke sequel with localized loss of strength and aphasia; Parkinson’s disease in a severe or unstable stage with motor skills, speech or affectivity impairments, as it would make it impossible to perform the evaluations.

For data collection, ten interviewers from the health area were selected, who underwent training and approach on ethical research issues. It is emphasized that the interviewers were followed-up until they demonstrated the necessary skills to apply the instruments for data collection.

Cognitive decline was assessed using the Mini Mental State Examination, considering the following cutoff points: ≤ 13 for illiterate people, ≤ 18 for low (1 to 4 incomplete years) and medium schooling (4 to 8 incomplete years), and ≤ 26 for high schooling (≥ 8 full years)^(^ 12 ^)^. The sociodemographic data and the morbidities were obtained through the application of a structured questionnaire.

The sociodemographic variables were age, gender, individual monthly income, housing arrangement, marital status and years of study. The clinical variables included were number of morbidities, depressive symptoms and impaired components of the frailty phenotype. To verify the depressive symptoms, the Abbreviated Geriatric Depression Scale validated in Brazil was used^(^ 13 ^)^.

The frailty syndrome was identified using the five components of the frailty phenotype^(^ 14 ^)^: (1) unintentional weight loss; (2) self-reported exhaustion and/or fatigue; (3) decreased muscle strength; (4) slow gait speed; (5) low level of physical activity.

Unintentional weight loss was assessed by the following question: In the last year, have you unintentionally lost more than 4.5 kg or 5% of your body weight? The self-report of exhaustion and/or fatigue was measured by two questions (items 7 and 20) of the Brazilian version of the depression scale of the Center for Epidemiologic Studies (CES-D). The older adults with a score of two or three in any of the questions met the frailty criterion for this item^(^ 15 ^)^.

To assess the decrease in muscle strength, the hand grip strength (HGS) was used, measured by a Jamar manual hydraulic dynamometer, Saehan® model (SH500 – 973), following the recommendations of the American Society of Hand Therapists. Three measures were obtained, presented in kilogram/force (kgf), with an interval of one minute between them, being considered the mean value. The following cutoff points adjusted for gender and Body Mass Index (BMI) were adopted: men (BMI ≤ 24 and HGS ≤ 29; BMI 24.1 – 26 and HGS ≤ 30; BMI 26.1 – 28 and HGS ≤ 30; BMI > 28 and HGS ≤ 32), and women (BMI ≤ 23 and HGS ≤ 17; BMI 23.1 – 26 and HGS ≤ 17.3; BMI 26.1 – 29 and HGS ≤ 18; BMI > 29 and HGS ≤ 21)^(^ 14 ^)^.

Regarding slowness in the gait speed, the walking time (in seconds) was considered. The older adult covered a total distance of 8.6 meters, the initial two meters and the final two meters being disregarded for calculating the time spent walking. Three measurements were taken, presented in seconds, considering the mean value. To this end, a Vollo® professional stopwatch model VL-1809 was used as standard, and the following cutoff points adjusted for gender and height were considered: men (Height ≥ 173 cm and Time ≥ 7 seconds; Height > 173 cm and Time ≥ 6 seconds), and women (Height ≥ 159 cm and Time ≥ 7 seconds; Height > 159 cm and Time ≥ 6 seconds)^(^ 14 ^)^.

To measure the level of physical activity and sedentary behavior, the long version of the International Physical Activity Questionnaire (IPAQ) was used, adapted for older adults^(^ 16 ^)^. The low level of physical activity was considered from a weekly time of 0 to 149 minutes of weekly physical activity of moderate to vigorous intensity^(^ 17 ^)^. In relation to sedentary behavior, the total time in a seated position, minutes/day, was determined by means of the weighted mean of the time in a seated position on a weekday and during the weekend. The longer the time, the greater the sedentary behavior^(^ 18 ^)^.

Regarding functional disability, the BADLs, IADLs and AADLs were evaluated. The BADLs were measured using the Katz Index adapted to the Brazilian reality^(^ 19 ^)^. For the IADLs, the Lawton and Brody Scale adapted in Brazil^(^ 20 ^)^ was applied. The AADLs were verified using the Scale of Advanced Activities of Daily Living, validated in Brazil, which includes 13 questions of a social nature^(^ 21 ^)^. For data analysis, the performance in the activities in each of the scales was considered, with higher scores for the BADLs and lower scores for the IADLs indicating greater dependence. For the AADLs, lower scores indicated less participation in the activities.

An electronic database was built using the Excel® program, with double typing. Inconsistencies were verified between the two databases, and corrections were performed when necessary. The analyses were conducted using the Statistical Package for Social Sciences (SPSS®) software, version 24, and Analysis of Moment Structures (AMOS®), version 24.

**Figure 1:**
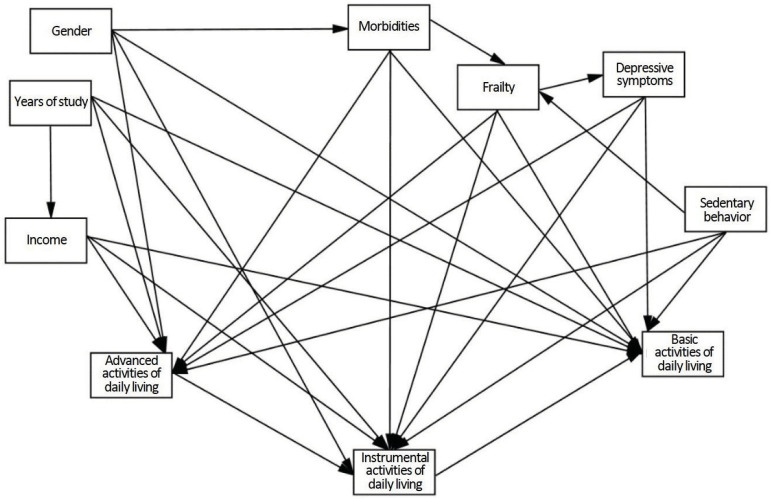
Hypothetical model tested for each age group

To analyze functional disability and its associated factors among community-dwelling older adults in the age groups from 60 to 69, 70 to 79, and 80 years old or more, trajectory analysis^(^ 11 ^)^ was used, which makes it possible to identify the estimates of the direct and indirect effects of the sociodemographic, economic, clinical and behavioral variables in the BADLs, IADLs and AADLs. In this sense, a hypothetical model was elaborated (Figure 1), composed of observed variables, represented by rectangles, and classified as endogenous and exogenous. The endogenous variables are those that receive directional arrows and measurement errors are attributed, specified by “e” in the models^(^ 11 ^)^.

Based on the specified hypothetical model, the following stages to analyze structural equations were conducted^(^ 11 ^)^: data collection, estimation of the model, and evaluation of the adjustment quality. The parameters were estimated using the Maximum Likelihood method and the fit quality of the models was assessed according to the Chi-square test (β^2^) p > 0.05; Goodness of Fit Index (GFI) ≥ 0.95; Comparative Fit Index (CFI) ≥ 0.95; Tucker-Lewis Index (TLI) ≥ 0.90; and Root Mean Error of Approximation (RMSEA) ≤ 0.05^(^ 11 ^)^. Initially, the hypothetical model test was carried out for each age group, and later the re-specifications were carried out. For that, the statistically non-significant trajectories were eliminated and the calculations of the modification indexes were performed^(^ 11 ^)^.

In the models analyzed, the direct effects were presented through the estimates of the standardized coefficients of the trajectories between the sociodemographic, economic, clinical and behavioral variables and the BADLs, IADLs and AADLs. In addition, the indirect effects (mediation effects) were determined from the intermediate paths between the aforementioned variables. In all tests, the type I error was set at 5% (p-value < 0.05).

The project was approved on May 9th, 2017, by the Research Ethics Committee, protocol No. 2,053,520. The older adults were presented with the objectives and the Free and Informed Consent Form and offered the relevant information. After the older adult’s consent and signing of the Form, the interview was conducted following the precepts established by Resolution 466/12 of the Ministry of Health.

## Results

In the current study, a total of 1,659 older adults were interviewed, of which 24 presented cognitive decline. Thus, the sample consisted of 1,635 older adults distributed in the age groups from 60 to 69 years old (n=688), 70 to 79 years old (n=627), and 80 years old or more (n=320).

The female gender presented a higher percentage for the three age groups, being 67.8% (IC95%: 64.3%-71.3%) for 60 to 69 years old, 64.4% (IC95%: 60.6%-68.1%) for 70 to 79 years old and 64.7% (IC95%: 59.3%-69.8%) for 80 years old or more. The individual income of a minimum wage was higher for the older adults aged 60 to 69 years old (44.1%; IC95%: 40.4%-47.8%, 70 to 79 years old (55.2%; IC95%: 51.3%-59.0%), and 80 years old or more (54.1%; IC95%: 48.6%-59.5%). Regarding the housing arrangement, 83.8% (IC95%: 81.0%-86.5%) of the older adults aged between 60 and 69 years old, 77,2% (IC95%: 73.8%-80.4%) between 70 and 79 years old, and 76,2% (IC95%: 71.3%-80.6%) aged 80 years old or mores, reported living with someone. Regarding marital status, those who had a partner for the age groups of 60 to 69 years old (54.0%; IC95%: 50.3%-57.7%) and 70 to 79 years old (42.7%; IC95%: 38.9%-46.6%) predominated, and widowers among those aged 80 and over (63.4%; IC95%: 58.1%-68.6%).

The mean schooling level was 5.23 (standard deviation 4.18) years among those aged 60 to 69 years old; 3.58 (standard deviation 3.48) for the older adults aged 70 to 79 years old, and 3.30 (standard deviation 3.59) in the age group of 80 years old or more.

The descriptive measures of the clinical and behavioral variables and of functional disability, included in the model according to age groups, of the older adults living in the health macro-region, are shown in Table 1.

**Table 1:** Distribution of the descriptive measures of the clinical and behavioral variables and of functional disability included in the model, according to age groups, of the older adults living in the health macro-region. Minas Gerais, Brazil, 2018

Variables	60 to 69		70 to 79		80 or more	
	Mean	Standard deviation	Mean	Standard deviation	Mean	Standard deviation
**Morbidities**	5.75	3.31	6.38	3.31	6.29	3.27
**Depressive symptoms**	3.32	3.17	3.53	3.18	3.66	2.87
**Frailty**	1.30	1.21	1.60	1.27	2.26	1.38
**Sedentary behavior**	280.71	138.92	318.45	161.35	353.54	181.52
**Basic Activities of Daily Living**	0.03	0.26	0.08	0.35	0.17	0.58
**Instrumental Activities of Daily Living**	18.99	2.54	18.09	3.00	16.03	3.91
**Advanced Activities of Daily Living**	5.75	2.29	5.20	2.41	4.55	2.25

**Figure 2:**
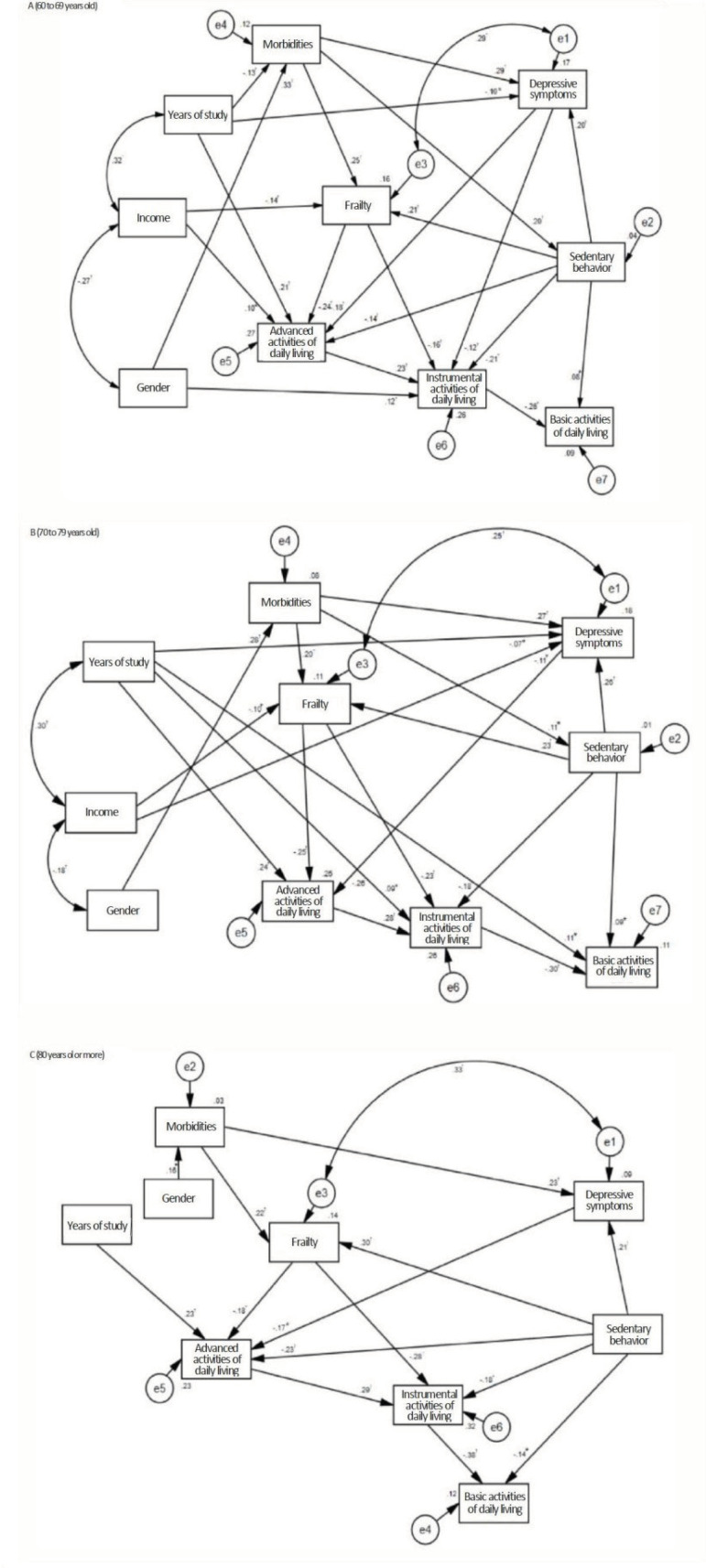
Models for analyzing the association between sociodemographic, clinical and behavioral variables with functional disability, according to age groups, of the older adults living in the health macro-region. Minas Gerais, Brazil, 2018

In the three age groups analyzed, it was verified that functional capacity for the AADLs was directly associated with the IADLs, which was directly associated with the BADLs (Figure 2). Additionally, for the three age groups, it was verified that functional capacity for the AADLs had an indirect association with the BADLs, mediated by the IADLs.

In the three groups, the lower level of schooling and the higher number of compromised components of the frailty and depressive symptoms phenotype were directly associated with lower participation in the AADLs (Figure 2). In addition, there were indirect associations between the highest number of morbidities and the lowest participation in these activities, mediated by frailty and depressive symptoms.

Among the older adults aged between 60 and 69 years old, lower income and higher sedentary behavior were directly associated with lower participation in the AADLs (Figure 2A). Furthermore, an indirect association was identified between the higher number of morbidities and the lower participation in these activities, mediated by sedentary behavior. For the older adults aged between 70 and 79 years old, frailty and depressive symptoms mediated associations between lower income and higher sedentary behavior with less participation in the AADLs. However, among those aged 80 or over, greater sedentary behavior was directly associated with less participation in these activities (Figure 2C).

In the three age groups, the greater number of compromised components of the frailty phenotype and sedentary behavior were directly associated with greater dependence for the IADLs (Figure 2). An indirect association was also observed between the higher number of morbidities and the greater dependence for the instrumental activities, mediated by frailty.

In the age group from 60 to 69 years old, female gender and higher number of depressive symptoms were directly associated with higher dependence for the IADLs (Figure 2A), while higher number of morbidities was indirectly associated with higher dependence for these activities, mediated by depressive symptoms and sedentary behavior. Lower schooling was indirectly associated with greater dependence for the IADLs, mediated by depressive symptoms, as well as lower income mediated by frailty.

Among the older adults aged between 70 and 79 years old, lower level of schooling was directly associated with greater dependence for the IADLs (Figure 2B). In the 60-69 and 70-79 age groups, greater sedentary behavior was directly associated with greater dependence for the BADLs (Figures 2A and 2B). For these two age groups, an indirect association between greater number of morbidities and greater dependence for the basic activities was also identified, mediated by sedentary behavior. For the older adults aged between 70 and 79 years old, higher schooling was directly associated with greater dependence for the BADLs (Figure 2B), and sedentary behavior, among those aged 80 years old or more (Figure 2C).

## Discussion

In the current study, functional disability occurred hierarchically. When considering the direct associations verified for the three age groups, the sociodemographic and clinical variables predominated in the functional incapacity for the AADLs, whereas the clinical and the behavioral predominated in the functional incapacity for the IADLs and sedentary behavior for the BADLs.

According to the results of the current research, there is a progressive loss of functional capacity, in which dependence for the AADLs is directly associated with the IADLs, and these, with the BADLs. In this sense, these findings confirm that the decline in functional capacity occurs hierarchically^(^ 22 ^-^ 23 ^)^, in which the older adults first have difficulties in performing activities that require greater complexity, independence and social participation^(^ 21 ^)^, later for those related to commitments and/or daily tasks^(^ 20 ^)^ and, finally, self-care activities^(^ 19 ^)^. This process is considered natural for aging; however, it can be delayed by adopting a healthy lifestyle and dependent on the sociodemographic and health conditions^(^ 24 ^)^.

However, in the study among Chinese people, it was observed that the AADLs do not seem to impose greater demands on the executive functions, which coordinate cognitive, emotional and motor activities during the performance of new and complex tasks^(^ 25 ^)^. This fact reiterates the relevance of identifying the factors directly related to the loss in the advanced activities, in order to rethink strategies that aim to postpone its appearance in order to favor the maintenance of the ADLs at the three levels for the longest possible time.

The lower participation in the advanced activities can be related to several factors, such as depressive symptoms in the older adults^(^ 26 ^)^; however, in the survey carried out among people aged 60 or over in Paraíba, no such association was found^(^ 27 ^)^. Considering the findings of the current study and the scarcity of articles on this theme, especially addressing the impact on the AADLs, it is suggested that early intervention in older adults with depressive symptoms should focus on these activities in search for preventing social isolation^(^ 26 ^)^. It is noteworthy that the activities must be rethought jointly with the older adult and/or the family members, prioritizing those that are of interest to them.

Regarding frailty as a factor associated with less participation in the advanced activities, the scientific literature reveals that this variable can be associated with sarcopenia^(^ 28 ^)^. Thus, such aspects must be investigated among community-dwelling older adults, in order to postpone and/or minimize the impact on functionality. In this sense, it is suggested that the health services be screened for frailty conditions in the aged population.

Among the older adults participating in the Frailty in Brazilian Older Adults (*Fragilidade em Idosos Brasileiros*, FIBRA) study conducted in Belo Horizonte, the prevalence of sarcopenia was 66.7% of the population partially dependent for the advanced activities^(^ 29 ^)^. It is noted that, in a research study conducted in Japan, the physical function, represented by handgrip strength and time in the physical performance test, was better among the individuals who performed AADLs^(^ 30 ^)^. Thus, preventive measures such as the tracking of factors related to functional loss to perform these activities, can subsidize health actions directed at the aged population.

In addition to that, it is relevant to pay attention to the factors that represent an indirect association with loss of functionality, such as the number of morbidities, which can impact on the level of frailty considering its physical aspect. In addition, there is the psychological impact, which can favor the increase in depressive symptoms, as observed in the model obtained in the current study.

According to research, there is evidence that older adults who maintain functional capacity for the advanced activities have fewer morbidities^(^ 31 ^)^. In this sense, it is also relevant to reflect on the directionality of this relationship, since functional loss can contribute to the increase in the number of diseases. Engagement in social activities can contribute to improving health status, which in turn favors social participation^(^ 32 ^)^.

As for the specificities related to the age group in social activities, it was observed in a study that the social involvement of the older adults is influenced by health conditions and functional capacity, as well as the assignment of gender and age roles and socioeconomic variables^(^ 27 ^)^, which can justify the difference in the mediators with less participation in the AADLs obtained in this research when stratified by age group. This fact favors interventions aimed at the specificities of each age group, being able to establish assertive measures, according to the context presented.

Sedentary time also stood out as a mediator of the relationship between morbidities and advanced activities; thus, it is understood that the greater the number of diseases, the older adults become less physically active, causing functional loss for performing such activities. According to a study, there is evidence of the need for interventions to encourage increased physical activity, as the frequency of interruptions in sedentary behavior was associated with a lower risk of impairment in the ADLs and physical dependence^(^ 33 ^)^, corroborating with the current survey. The identification of modifiable behaviors associated with the maintenance of the ADLs is relevant for the development of prevention strategies aimed at independent life in older adults^(^ 33 ^)^.

With regard to the instrumental activities, the dependence associated with the frailty syndrome was verified in national^(^ 34 ^-^ 37 ^)^ and international studies^(^ 38 ^)^. Considered as public health problems, the frailty syndrome and functional dependence represent a challenge for societies in general, as they have a negative impact on the health and quality of life of the aged population^(^ 36 ^)^. Therefore, the frequent monitoring of the older adults, both frail and pre-frail, by health professionals, must be a priority for the preservation of autonomy and independence^(^ 34 ^)^. This can be achieved through the prevention and control of the risk factors that precede the onset of the frailty syndrome^(^ 35 ^)^.

Sedentary behavior, as well as frailty, had a negative effect on functional capacity for the instrumental activities, in which the longer the time in a seated position, the worse the performance in daily tasks, as identified in national^(^ 39 ^)^ and international studies^(^ 6 ^)^. These results are relevant, considering that, in systematic reviews, it was identified that people aged 60 years old or older spend a mean of nine hours a day in sedentary behavior^(^ 40 ^)^ and, as a consequence, present worse health conditions, which represent a risk not only to life, but also to the preservation of the autonomy and independence of the aging population^(^ 41 ^)^.

The indirect association between the higher number of morbidities and dependence for the IADLs, mediated by frailty, can be explained by the fact that chronic non-communicable diseases (CNCDs) and functional disabilities are more frequent with advancing age^(^ 1 ^)^, and are related to the development of the frailty syndrome^(^ 36 ^)^. In studies carried out among community-dwelling older adults, it was verified that polymorbidity was associated with functional dependence for carrying out activities related to commitments and/or daily tasks^(^ 42 ^-^ 43 ^)^; for this reason, preventing these conditions through the necessary support for each individual must be the main objective of the health systems. Recognizing the factors that can compromise the functional capacity of the older adults enables the planning of health actions directed to the needs of this age group^(^ 43 ^)^.

The female gender associated with functional disability for the IADLs is consistent with studies carried out in communities in the Northeast^(^ 31 ^)^ and South of Brazil^(^ 42 ^)^, as well as in a research study conducted in Japan, in which it was observed that, among the younger aged individuals, functional dependence for these activities was higher in females^(^ 24 ^)^, which may negatively affect their autonomy and social life^(^ 31 ^)^. Furthermore, individuals with impaired functional conditions are more vulnerable to the frailty syndrome, violence and maltreatment, and to institutionalization^(^ 31 ^)^. Thus, health professionals must guide their care process in the specific needs of the older adults, considering the differences between the genders, in search for preventing functional disability and/or minimizing its consequences.

The worse performance in the instrumental activities associated with a greater number of depressive symptoms, among the older adults aged 60 to 69 years old, is consistent with data from national^(^ 27 ^,^ 37 ^)^ and international studies^(^ 6 ^)^. Depression is now considered an important predictor of disability, with triggering and worsening of functional decline^(^ 44 ^)^. The need for the health professionals to identify depressive symptoms in these individuals must be emphasized, as it is still underdiagnosed and considered as a natural consequence of aging^(^ 44 ^)^. In view of the population aging process, it is necessary to: implement strategies to welcome the older adults, strengthen the health professional/older adult/family bond, early identify factors that may contribute to the worsening of the depressive symptoms such as, for example, functional limitations that can harm the well-being of this population^(^ 27 ^,^ 37 ^)^.

In the same age group, depressive symptoms also mediated the association between the number of morbidities and functional dependence in the instrumental activities. In this perspective, in the research study carried out among older adults with polymorbidity, it was verified that the prevalence of the indicative of depressive symptoms was twice as high in this group in relation to those without this condition^(^ 45 ^)^, demonstrating the impact of the CNCDs on the mood of the aged population, which has the negative consequences of functional decline^(^ 6 ^,^ 27 ^,^ 37) and of an increased demand for the health services and higher risk of mortality^(^ 3 ^)^. Therefore, the early identification of these factors is essential for the prevention of functional disability and the implementation of public policies to strengthen social and family ties, creating support networks for active and healthy aging^(^ 45 ^)^.

Another mediating variable of the aforementioned association was sedentary behavior. According to previous research studies, the older adults who spend more time in a seated position have worse health conditions^(^ 6 ^,^ 41 ^)^. In addition, the higher incidence of CNCDs is related to functional decline^(^ 42 ^-^ 43 ^)^. Thus, the proposals for support and treatment for the aged population must be more directed towards determining the changes imposed by the chronic health conditions on functional capacity than the simple presence of morbidities^(^ 42 ^)^. The health professional, especially the nurse, through gerontological consultation and home visits, can identify the older adults with potential risks of functional decline, enabling the proposal of interventions and preventive measures.

Corroborating with the current research, in national studies among community-dwelling older adults, it was observed that functional disability for the instrumental activities was greater among those with lower schooling^(^ 37 ^,^ 46 ^)^ and with depressive symptoms^(^ 37 ^)^. The activities related to commitments and/or daily tasks require cognitive skills that can be influenced by low schooling^(^ 37 ^,^ 46 ^)^; thus, low schooling level can contribute to greater vulnerability in the older adults^(^ 47 ^)^. In this context, it is essential that the health professionals consider this factor, which can interfere with the understanding of information and adherence to the necessary care actions to maintain functional independence.

The association between the lower income of younger older adults and worse performance in the instrumental activities, mediated by frailty, can be explained by the fact that, in addition to contributing to insufficient social and health resources and poor self-care, low income is also related to frailty^(^ 14 ^,^ 34 ^)^, since this condition can favor and reflect on the lower degree of physical and psychological well-being and, consequently, on greater functional dependence^(^ 14 ^,^ 34 ^)^.

Data similar to the current study was identified in a research study carried out in the community, in which the older adults with lower schooling and who did not know how to read had greater functional dependence for the IADLs^(^ 37 ^)^, similarly to what was observed in the multicentric research study with the Brazilian aged population^(^ 48 ^)^. This result is justified by the fact that these activities require a higher level of literacy from the older adults, since they have a certain degree of difficulty for their complete execution^(^ 48 ^)^.

Regarding the BADLs, the association between the greater time spent in sedentary behavior and the inability to perform them, among the younger older adults, can be explained by the components of physical fitness. A study has shown that older adults with longer time spent in sedentary behaviors perform worse in the components of physical fitness^(^ 49 ^)^, which are decisive for activities related to self-care^(^ 50 ^)^. In addition, sedentary behavior has been shown to be associated with worse outcomes for inflammatory biomarkers^(^ 39 ^)^.

The time spent in sedentary behavior was also considered, for older adults from 60 to 69 years old, as a mediator of the association of the number of morbidities with the disability for the basic activities. Previous studies have shown an association between sedentary behavior and CNCDs, with older adults with multimorbidities being more sedentary^(^ 51 ^-^ 52 ^)^. Regarding the association of sedentary behavior with functional disability for self-care activities, in a systematic review study with meta-analysis, a positive relationship was identified between functional disability and a longer mean time of sedentary behavior in older adults^(^ 53 ^)^. This reinforces the need to develop interventions aimed at preventing sedentary behavior in this age group, especially among younger older adults.

The research has as limitations the measure of the time spent in sedentary behavior and physical activity by self-report, and the non-inclusion of variables related to lifestyle, social participation and personal and environmental factors, which can act as barriers or facilitators in carrying out the activities of daily life. However, the findings allow for an expanded understanding of the factors associated with functional disability in older adults. It was observed that, regardless of age, low schooling, frailty, depressive symptoms and sedentary behavior were directly related to functional disability for the ADLs; and that they must be considered in the elaboration of health care strategies for the older adults, aimed at preserving autonomy and independence.

Thus, the results provide subsidies for further research studies, and it is suggested to carry out multicenter studies and national surveys, with representative samples of the aged population in different Brazilian states, in order to contribute to improving health care for the older adults.

## Conclusion

For the three age groups analyzed, it was verified that functional disability occurs hierarchically. Taking into account the direct associations found for the three age groups, the sociodemographic and clinical variables predominated in functional disability for the advanced activities, whereas the clinical and behavioral variables predominated in functional disability for the instrumental activities, and sedentary behavior for the basic activities.

These data contribute to directing the health care of the older adults, since it was possible to identify the factors at work in the functional disabilities, highlighting the direct and indirect relationship variables, aspects that had not yet been described in the scientific literature.

In this sense, research studies on the health conditions of the older adults are relevant, aiming to establish follow-up actions in the health services to postpone the negative impact on functionality. In addition to that, primary care nurses are the professionals with the greatest contact with the community-dwelling older adults. Therefore, the identification of habits and behaviors helps to reflect on the need to develop actions that enable the adoption of measures to improve active time, considering the impact of sedentary behavior.
